# A knowledge, attitude, and practice survey on mediation among clinicians in a tertiary-care hospital in Singapore

**DOI:** 10.1371/journal.pone.0199885

**Published:** 2018-07-09

**Authors:** Wenrong Lin, Zheng Jye Ling, Siqi Liu, Joel Tye Beng Lee, Marcus Lim, Aloysius Goh, Sean Lim, Peter George Manning, Mengling Feng, Tze Ping Loh

**Affiliations:** 1 Legal Office, National University Hospital, Singapore; 2 Academic Information Office, National University Hospital, Singapore; 3 Singapore International Mediation Institute, Singapore; 4 Peacemakers Consulting Services Pte Ltd, Singapore; 5 Clinical Risk Management, National University Hospital, Singapore; 6 Saw Swee Hock School of Public Health, National University of Singapore, Singapore; 7 Department of Laboratory Medicine, National University Hospital, Singapore; 8 Biomedical Institute for Global Health Research and Technology, National University of Singapore, Singapore; Medical University Graz, AUSTRIA

## Abstract

Healthcare delivery is a highly complex, deeply personal and costly endeavour that involves multiple specialties and services. There is an imbalance in knowledge between the healthcare provider and consumer that may contribute to doubts and uncertainty over treatment and outcomes. It is unsurprising that conflict and dispute can develop between healthcare providers and patients and their next-of-kin. The use of mediation in the healthcare setting has recently been promoted in many developed countries, including Singapore. We administered a detailed 32-item survey in a large tertiary-care teaching hospital to improve our understanding of the knowledge, attitude and practice of dispute resolution among clinicians to pave the way for better strategies to improve the adoption of mediation in healthcare setting. Ninety-seven respondents had an average of 62% (SD: 12%) knowledge score. The most common misconceptions held by the respondents about mediation were: (1) mediation was about fact-finding, (2) mediation is limited to only certain types of dispute, (3) mediation proceeds by both parties giving their account of the dispute, then a third party decides a settlement, (4) the average time it takes to resolve a dispute through mediation, (5) the cost of mediation, (5) the venue of mediation, (6) the person determining the outcome of mediation, (7) confidentiality of mediation. In general, the respondents were positive about the use of mediation as a dispute resolution tool. When asked to indicate the relative importance of different outcomes of dispute resolution, financial compensation and waiver of hospital bill attracted mixed responses while understanding facts of dispute, assurance that the same error would not recur, and offering corrective treatment were rated as being important. By contrast, seeking an apology from the complainant was considered neutral to somewhat important and the respondents were least concerned with the publicity of the dispute. Direct negotiation with the complainant was considered the most time- and cost-efficient means of resolving a dispute while the opposite was true for litigation. Mediation was considered the approach where the clinicians are most likely to achieve their desired outcome while litigation was considered least likely to produce a favourable outcome. Approximately half of the respondents reported having personal experience or known of a colleague who had been involved in a medico-legal dispute. A quarter of these cases were resolved by direct negotiations with the complainant while lawyers, the judge and mediation, resolved approximately 15% each, respectively. The knowledge base of the clinicians in this study about mediation was moderate and probably reflected the general lack of direct experience in the resolution of a dispute or training in mediation. This further corroborated with the general response that the uptake of mediation in the healthcare setting is currently poor in Singapore due to the lack of awareness and perceived lack of avenue among the surveyed clinicians. Any further work to be done with clinicians may be in the direction of (1) increasing general understanding of mediation, (2) increasing awareness of avenues for mediation, and (3) becoming better aware of when to propose mediation.

## Introduction

Healthcare delivery is a highly complex and costly endeavour that involves multiple specialties and services. It is also deeply personal and often evokes strong emotions not only in the patient, but also the caregivers. Moreover, there is an imbalance in knowledge between the healthcare provider and consumer that may contribute to doubts and uncertainty over treatment and outcomes [1,2]. It is unsurprising that conflict and dispute can develop between healthcare providers and patients and their caregivers.

There is an increasing number of disputes in the healthcare setting being sent for litigation [3,4]. More recently, open communication has been advocated as a means to reduce the occurrence of dispute in the healthcare setting [5]. Open communication is a practice of honest disclosure to the patient and caregivers when things do not happen as expected. Open communication can resolve a dispute before it reaches the litigation stage. Nevertheless, there are still disputes that cannot be fully resolved by open communications. For these cases, alternate dispute resolution strategies have been gaining traction. Among the alternate dispute resolution strategies, mediation is an important tool to achieve an amicable settlement that is acceptable between the parties [6–8].

The use of mediation in the healthcare setting has recently been promoted in many developed countries, including Singapore. We undertook a detailed survey in a large tertiary-care teaching hospital to improve our understanding of the knowledge, attitude and practice of dispute resolution among clinicians to pave the way for better strategies to improve the adoption of mediation in healthcare setting.

## Materials and methods

The National University Hospital, Singapore is a 1100-bed tertiary-care teaching public hospital serving the Western half of Singapore. It is affiliated to the National University of Singapore and provides a complete suite of medical and surgical services that encompasses obstetrics and gynaecology, paediatrics surgery and medicine, adult surgery and medicine, and orthopaedics. We received ethics approval for this study (National Healthcare Group Domain Specific Review Board, reference number: 2016/01362).

We developed a 32-item questionnaire that probed the knowledge, attitude and practice (based on past experience as well as hypothetical scenario) of mediation and dispute resolution of the clinicians through a mix of open- and close-ended questions (Table 1). The respondents were also asked to indicate their preferences using Likert-scale and ranking. The complete questionnaire is provided alongside the results in the following section. The survey questionnaire was developed by the investigators and piloted on a small group of 7 respondents to refine and improve it. Once finalised, the questionnaire was adopted on the Google survey platform. An email invitation was sent to all the clinicians in our institution (n = 1264) with a reminder email sent one and three months later. Additionally, the invitation to participate in the questionnaire survey was placed on screen before the weekly grand round in our hospital for four months. The survey participants provided informed consent electronically prior to undertaking the questionnaire survey.

The respondents voluntarily participated in the survey and provided implied consent to have their data analysed in an anonymised, aggregated manner. The survey did not collect personal or identifiable data.

### Statistical analysis

The survey responses for each question were summarised using simple descriptive statistics. The percentage of the response was calculated with the total number of participants (i.e. n = 97) as the denominator. Subsequently, the participants were then divided into those who had prior experience in managing conflict and those who had not. The responses to the knowledge and attitude questions (i.e. Questions 1 to 17) between the two groups of participants were compared using Fisher's exact test (two-tailed).

The knowledge of the participants was also scored against idealised/preferred answers for Questions 1 to 13, which were determined by the investigators. For questions with only one 'model' answer, a matched response was given a score of 1 while any other response was scored 0. For questions with multiple preferred answers, the respondents were scored 1 if they have fully matched responses, 0.5 if they had partially matched responses and 0 if they have fewer than half matched responses. The scores were then expressed as a percentage of the maximum of 13. The knowledge scores were correlated with the preferences of the clinicians for Questions 14, 15 and 20 in the Attitude section. A p-value of <0.05 was considered significant in this study.

## Results

In total, we received 97 responses from the clinicians, representing a response rate of 8.7% from 1121 eligible clinicians in our hospital. Nearly two-thirds of the respondents were senior staff who had more than 6 years of working experience (65/97, 67%), followed by junior staff who had 0–3 years of working experience (19/97, 20%) and those with 4–6 years of experience (13/97, 13%). Correspondingly, 62 (64%) clinicians had postgraduate qualifications. Just less than half (45/97, 46%) of the clinicians were female.

The responses to the individual questions are summarised in Table 1 below. The preferred/ idealised responses for the knowledge-related questions are highlighted in *italic*.

**Table 1:** Summary of the responses to the survey questions.

Knowledge 1. Mediation is a process to ____________. (Choose more than one if needed)	
A: Find out the facts about a dispute 53 (54%)	D: Decide on monetary compensation 12 (12%)
B: Decide on who was at fault 5 (5%)	E: Get someone to apologise 4 (4%)
*C*: *To settle a dispute with the help of a neutral third party* 91 (94%)	F: Set expectations of complaining party 32 (33%)
2. What are the types of dispute that can be resolved through mediation? (Choose more than one if needed)	
*A*: *Financial bill* *49 (51%)*	D: Dissatisfaction with meals/facilities in hospital 58 (60%)
*B*: *Dissatisfaction with medical care* *91 (94%)*	Others: All of the above, anything 2 (2%)
*C*: *Dissatisfaction with attitude of healthcare worker* *83 (86%)*	
3. Typically, who is involved in a mediation process?	
*A*: *The patient and/or their friend/family members* 88 (91%)	D: Lawyers 16 (16%)
*B*: *Hospital administrative staff* 66 (68%)	E: Judge 1 (1%)
*C*: *Hospital staff involved in the care of patient (e*.*g*. *doctors*, *nurses)* 83 (86%)	*F*: *A person specially trained to assist dispute resolution (mediator)* 89 (92%)
4. Typically, how is mediation conducted?	
A: The person who initiated the mediation will give his account of the dispute, then makes demands to settle dispute 0 (0%)	*C*: *Both parties give their account of the dispute*, *then negotiate for a settlement* *57 (59%)*
B: The person who is being complained against gives his account of the dispute, then makes offers to settle dispute 1 (1%)	D: Both parties give their account of the dispute, then a third party decides a settlement 39 (40%)
5. On average, how long do you think mediation will take to settle a dispute?	
A: Half a day 13 (13%)	C: 3–7 days 27 (28%)
*B*: *1–2 days* 16 (16%)	D: >7 days 41 (42%)
6. On average, how much do you think it costs in total to mediate a dispute?	
A: S$100–500 17 (18%)	*C*: *S$1001–5000* 42 (43%)
B: S$501–1000 17 (18%)	D: S$5000 or more 21 (22%)
7. Where is mediation usually conducted?	
A: In the court 4 (4%)	D: A third party venue 24 (25%)
B: In the hospital 20 (21%)	*E*: *Anywhere both parties agree* 49 (51%)
C: In the house of the patient 0 (0%)	
8. Typically, who decides on the final outcome of the mediation?	
A: The person complaining 2 (2%)	D: Judge 0 (0%)
B: The hospital 2 (2%)	E: A person specially trained to assist with dispute resolution (mediator) 43 (44%)
C: Lawyers 1 (1%)	*F*: *The person complaining and the hospital staff (both parties)* 49 (51%)
9. Is it compulsory to mediate a healthcare-related dispute before going to court in Singapore?	
Yes 33 (34%)	*No* 64 (66%)
10. Do you think information revealed during mediation is confidential (kept secret)?	
*Yes* 87 (90%)	No 10 (10%)
11. Can the information revealed in mediation be used in court later?	
Yes 66 (68%)	*No* 31 (32%)
12. Does initiating the mediation process mean you cannot go on to court later?	
Yes 2 (2%)	*No* 95 (98%)
13. Is the outcome of mediation final and you can’t go to court thereafter?	
Yes 7 (7%)	*No* 90 (93%)
**Attitude** 14. On a scale of 1 to 7, how do you rate the importance of mediation in dispute resolution? 1 = Totally unimportant. 7 = Very important.	

1	2	3	4	5	6	7
0 (0%)	0 (0%)	0 (0%)	1 (1%)	19 (20%)	30 (31%)	47 (48%)
15. On a scale of 1 to 7, how likely are you to use mediation to resolve a dispute? 1 = Very unlikely. 7 = Most likely.						
1	2	3	4	5	6	7
1 (1%)	0 (0%)	1 (1%)	7 (7%)	14 (14%)	27 (28%)	47 (48%)
16a. In the event of a dispute with a patient, on a scale of 1 to 7, please rate the importance of maintaining a good relationship with the patient / staff / hospital during dispute resolution. 1 = Totally unimportant. 7 = Very important, I want to maintain a good relationship						
1	2	3	4	5	6	7
0 (0%)	0 (0%)	0 (0%)	1 (1%)	5 (5%)	28 (29%)	63 (65%)
16b. Please rank the following items in order of importance as an outcome of dispute resolution (1 being least important, 4 being neutral, 7 being most important). Do not select any column more than once.						

Rank	1	2	3	4	5	6	7
Financial compensation	4 (4%)	19 (20%)	9 (9%)	19 (20%)	21 (22%)	16 (16%)	9 (9%)
Waiver of hospital costs	3 (3%)	13 (13%)	18 (19%)	13 (13%)	27 (28%)	16 (16%)	7 (7%)
An apology from the other party	1 (1%)	8 (8%)	4 (4%)	30 (31%)	15 (15%)	21 (22%)	18 (19%)
Understanding the facts of the event	4 (4%)	2 (2%)	5 (5%)	2 (2%)	10 (10%)	25 (26%)	49 (51%)
Assurance that the event will not happen again/to other patients	1 (1%)	7 (7%)	4 (4%)	5 (5%)	17 (18%)	29 (30%)	34 (35%)
Having corrective treatment/action to improve the condition	5 (5%)	5 (5%)	1 (1%)	4 (4%)	12 (12%)	32 (33%)	38 (39%)
Publicity about the event	42 (43%)	15 (15%)	8 (8%)	19 (20%)	7 (7%)	2 (2%)	4 (4%)
17. I am likely to get my desired outcome of dispute resolution through: (please rank the likelihood, 1 being least likely, 3 being most likely). Do not select any column more than once.							

Rank	1	2	3
Direct negotiation with patient / staff / hospital	14 (14%)	42 (43%)	41 (42%)
Mediation	0 (0%)	41 (42%)	56 (58%)
Going to court	66 (68%)	25 (26%)	6 (6%)
18. I am likely to spend the most time resolving a dispute through (please rank the likelihood, 1 being longest time, 3 being fastest resolution). Do not select any column more than once.			
Rank	1	2	3
Direct negotiation with patient / staff / hospital	12 (12%)	41 (42%)	44 (45%)
Mediation	5 (5%)	57 (59%)	35 (36%)
Going to court	81 (84%)	8 (8%)	8 (8%)
19. I am likely to spend most expenses resolving my dispute through (please rank the likelihood, 1 being most expensive, 3 being least cost). Do not select any column more than once.			
Rank	1	2	3
Direct negotiation with patient / staff / hospital	3 (3%)	27 (28%)	67 (69%)
Mediation	5 (5%)	67 (69%)	25 (26%)
Going to court	89 (92%)	5 (5%)	3 (3%)
20. On a scale of 1–7, please rate your interest in learning about ways to resolve disputes. (1 = will never bother, 4 = neutral, 7 = will certainly learn more)			

1	2	3	4	5	6	7
0 (0%)	1 (1%)	3 (3%)	5 (5%)	19 (20%)	34 (35%)	35 (37%)
21. On a scale of 1–7, please rate the importance of teaching mediation to hospital staff. (1 = not important at all, don't teach, 4 = neutral, 7 = very important, must teach)						
1	2	3	4	5	6	7
0 (0%)	1 (1%)	0 (0%)	4 (4%)	14 (14%)	34 (35%)	44 (45%)
**Practice** 22a. Have you ever managed any conflict/dispute from patient / patient’s family members / friends during your employment with the hospital?						

Yes 52 (54%)	No 45 (46%)
22b. If yes to question 22(a) above, please indicate an estimated number of cases managed.	

1	2	3	4	5	>5
3 (8%)	11 (28%)	13 (13%)	3 (33%)	12 (30%)	8 (20%)
23a. Have you received any training in relation to management of conflict/dispute in the hospital setting?					

Yes 32 (33%)	No 65 (67%)
23b. Would you like to receive any training in relation to management of conflict/dispute in the medical setting?	
Yes 82 (85%)	No 15 (15%)
24. On a scale of 1 to 7, how comfortable are you with handling the patient / patient’s family members / friends in relation to any negative experience / outcome? ____(1 = not comfortable at all, 4 = neutral, 7 = very comfortable)	

1	2	3	4	5	6	7
3 (3%)	10 (10%)	11 (11%)	17 (18%)	29 (30%)	20 (21%)	7 (7%)
25a. Have you been involved in any medico-legal dispute/complaint or know of hospital professionals with such experience?						

Yes (please complete 25b) 49 (51%)	No 48 (49%)
25b. If yes to question 25a above, was the dispute/complaint resolved through: (choose more than one if needed)	
A: Direct negotiation between the disputing parties 24 (25%)	D: Mediation 16 (16%)
B: Settled by lawyers 14 (14%)	E: Decided by SMC Complaints Committee 1 (1%)
C: Decided by court 15 (15%)	F: Uncertain 3 (3%)
For questions 26–30, please answer them in your experience as a patient or a patient’s next of kin. 26. Which party at the hospital will you speak to if you have concerns about your care (or your family member's care)? Select more than one if applicable.	
A: Attending doctor 77 (79%)	C: Head of Department/ Clinical Director 35 (36%)
B: Nurse 27 (28%)	D: Patient Relations Department 28 (29%)
27. Have you reported any negative experience/outcome about your care or your family's care (e.g. poor outcome) to the hospital? If yes, complete the questions below. If no, skip this section. a. What prompted you to do so?	

Family pressure	1
Intra-operation unexpected cardiac arrest	1
Poor cannulation and attitude from fellow staff who failed the cannulation	1
Poor quality of Care	1
Slowness in taking of parameters and setting up an IV drip in my mother after RTA	1
Things could have been done better by a more senior staff	1
Upset	1
b. Who did you report it to?	
A senior staff	1
Head of department	1
Nursing officer	1
Patient's relation department	1
Registrar of the ward (in a different hospital)	1
Sister of radiology department, who was in charge of her staff nurses	1
Supervisor of the involved person	1
c. What was the outcome?	
Apology	2
Appropriate actions taken	1
Investigation with formal reply	1
Not aware	1
Patient recovered without any morbidity	1
Senior staff took over, good outcome	1
Was the outcome satisfactory?	
Yes 4	No 2
d. What do you think will satisfy you?	
A sincere apology from the fellow staff directly	1
Better training and supervision for junior staff	1
Improved care	1
Reassurance that it will not be repeated	1
Resolution of issues	1
Sincerity from hospital	1
28. If you have had a negative experience/outcome but did not wish to raise it to the hospital, what stopped you from raising it?	

A: Relatively small issue 59 (61%)	E: Fear of repercussion / receive poor appraisal 12 (12%)
B: Want to maintain good relationship with staff / hospital 35 (36%)	Others: Takes too much time/effort 1 (1%)
C: Did not want to cause trouble to medical team 33 (34%)	Others: No harm to patient 2 (2%)
D: Lack of avenue to provide feedback 14 (14%)	Others: No experience 5 (5%)
29. How would you have preferred to resolve the negative experience/outcome? Please rate your preference: 1 = least preferred, 4 = neutral, 7 = most preferred.	

Rank	1	2	3	4	5	6	7
A neutral third party (e.g. another colleague)	1 (1%)	1 (1%)	5 (5%)	19 (20%)	29 (30%)	32 (22%)	10 (10%)
Raising it formally to relevant hospital authorities	4 (4%)	7 (7%)	19 (20%)	19 (20%)	20 (21%)	18 (19%)	10 (10%)
Seeking legal advice	35 (36%)	20 (21%)	10 (10%)	17 (18%)	6 (6%)	5 (5%)	4 (4%)
30. The uptake of mediation is low in the healthcare setting in Singapore. In your opinion, what is preventing people from using mediation as a way to resolve dispute?							

A: Lack of awareness, knowledge 75 (77%)	C: Perceived as time consuming 5 (5%)
B: Perceived lack of access 6 (6%)	D: Perceived as troublesome 3 (3%)
31. Do you know how to access mediation services in the healthcare setting?	
A: No, I do not know how to access mediation services 79 (81%)	C: Lawyer 2 (2%)
B: Patient relation/ clinical risk management 6 (6%)	D: Administrative staff 1 (1%)
32. What else would you have liked to know about mediation in the healthcare setting?	
A: Accessibility, avenue for advice 22 (23%)	D: Case examples and possible outcomes 7 (7%)
B: Process of mediation 10 (10%)	E: Who are the mediators and their qualifications 4 (4%)
C: Cost and who bears it 9 (9%)	F: Confidentiality of the mediation process and findings 3 (3%)

### Knowledge of the respondents

The respondents had an average of 62% (SD: 12%) knowledge score. The most common misconceptions held by the respondents about mediation were (see Questions 1 to 13): (1) mediation was about fact-finding, (2) mediation is limited to only certain types of dispute, (3) mediation proceeds by both parties giving their account of the dispute, then a third party decides a settlement, (4) the average time it takes to resolve a dispute through mediation, (5) the cost of mediation, (5) the venue of mediation, (6) the person determining the outcome of mediation, (7) confidentiality of mediation.

### Attitude of the respondents

In general, the respondents were positive about the use of mediation as a dispute resolution tool (Questions 14 and 15). The knowledge scores did not correlate with the inclination of the clinicians to learn or use mediation as a dispute resolution tool (correlation coefficient <0.3). An overwhelming majority (94%) of respondents felt that maintaining a good relationship with the complainant was important (Question 16a). When asked to indicate the relative importance of different outcomes of dispute resolution (Question 16b), financial compensation and waiver of hospital bill attracted mixed responses while understanding facts of dispute, assurance that the same error would not recur, and offering corrective treatment were rated as being important. By contrast, seeking an apology from the complainant was considered neutral to somewhat important and the respondents were least concerned with the publicity of the dispute.

Direct negotiation with the complainant was considered the most time- and cost-efficient means of resolving a dispute while the opposite was true for litigation (Questions 17–19). Mediation was considered the approach where the clinicians are most likely to achieve their desired outcome of dispute resolution (Question 17) while litigation was considered least likely to produce a favourable outcome.

### Practice of the respondents

Just over half of the respondents (54%) had experience managing a dispute with patients (Question 22). Of these, half had managed five or more disputes in the past. Interestingly, prior experience in managing dispute did not produce statistically different responses to the knowledge- and attitude-related questions (Questions 1 to 17). Nevertheless, the overall knowledge score was higher in respondents with experience managing a dispute with patients compared to those without such experience (65% vs 58%, p <0.005). Approximately half of the respondents reported having personal experience or known of a colleague who had been involved in medico-legal dispute (Question 25). A quarter of these cases were resolved by direct negotiations with the complainant while lawyers, the judge and mediation, resolved approximately 15% each, respectively.

When the clinicians were asked to put themselves in the shoes of the patients (or their next of kin), they mostly preferred to raise concerns about their care with a fellow clinician (Question 27). The clinicians would choose not to raise a negative experience with a hospital when they consider it as a minor issue (61%) or because they wish to maintain a good relationship with the colleague/ hospital (36%)(Question 28). They were somewhat likely to resolve the matter through a neutral third party or raising it to the hospital authority than considering legal advice (Question 29).

## Discussion

Traditionally, public healthcare institutes have in-build mechanisms to manage disputes. The majority of cases are handled by such quality service management mechanism, the “direct negotiation” route, where a hospital representative will investigate the complaints and respond to the patient or next-of-kin appropriately. Direct negotiation is generally the preferred tool that a healthcare institution uses to resolve dispute since it is important for the parties to establish direct communication to ascertain the details of the dispute. This tool is particularly useful if the communication/ content is straightforward, and there is no ill will between the parties and parties are clear about their positions. However, in the event of escalation, the dispute will proceed in the direction of litigation, where the insurers and lawyers of both the hospital and the clinician, will be activated to defend against the claims.

Mediation is a voluntary and confidential conflict resolution process where a neutral third party, the mediator, works alongside the parties to find mutually agreeable solutions to the dispute at hand. It is useful when the relationship between the parties is strained and direct communication has reached an impasse. It is also useful if one party is seeking non-monetary resolution (such as apology, acknowledgement, change in process), which the litigation process cannot provide for. The mediation process caters a confidential platform for parties to explain, exchange and clarify information, restore relationship/ trust. These are elements to an amicable settlement, or sets parties on the path to reach a sustainable and enforceable settlement. There are various accreditation schemes in Singapore to train and accredit mediators. Such training equips mediators with a framework and skillsets to facilitate discussion between disputing parties to achieve better communication, appreciate different perceptions, manage emotions, and overcome impasses. The mediators do not make any orders or decisions on the outcome.

The mediation movement in Singapore became relatively more active in the 1990s and since then the courts actively promote and encourage the use of mediation for suitable disputes [9]. The mediation movement for the healthcare industry came into spotlight in recent years. With increasing insurance premiums, a potential upward trend of medical litigation and more complex care required due to the aging population, the Ministry of Health Holdings set up its Mediation Unit in 2014 and re-introduced the Healthcare Mediation Scheme to encourage take up on mediation for healthcare-related disputes. Prior to that, the Singapore Medical Council (SMC) and the Singapore Mediation Centre piloted a SMC-Medical Council Mediation Scheme for SMC Complaints Committee to refer matters for mediation (Section 42(4)(b)(ii) of the Medical Registration Act). According to the Head of Mediation Unit, we note that to-date, Healthcare Mediation Scheme has since facilitated the mediation of 25 cases, of which 18 cases were settled, 6 were not, and 1 of them is pending negotiations. The settlement rate is approximately 75% (private communication by the Healthcare Mediation Unit at Ministry of Health Holdings Pte Ltd).

The Honourable the Chief Justice, Sundaresh Menon, highlighted in his speech of 28 October 2014 to the Obstetrical & Gynaecological Society of Singapore that (1) the tort of negligence and the adversarial nature of the litigation system do not provide a holistic solution for disputes relating to bad medical outcome; (2) an increase in medical malpractice law suits will lead to more clinicians practising defensive medicine and result in a lose-lose situation for both individuals and clinicians.

Identifying the main issues of disputes in healthcare industry as “dashed expectations and miscommunications”, mediation, which is a process that focuses on improving communication, active listening and extra-judicial resolutions, was proposed to be the first port of call for potential medical negligence disputes [9].

Some pertinent observations from this survey:

1. There is an expectation of mediation to go beyond 7 days (Question 5) and yet a large group shows willingness to use mediation to resolve dispute (Question 15).
2. Despite not having training in mediation, the majority of the surveyed participants showed above average knowledge of mediation.
3. Mediation may be a suitable platform for clinicians to explain the circumstances and treatment to the patients or their next-of-kind, as compared to litigation. This is because the clinicians surveyed had indicated that “understanding the facts of the dispute” as an important outcome of mediation. Aside from mediation, direct negotiation could also be explored as achieving this outcome, albeit without the presence of a moderating third party (mediator).
4. Most clinicians find that the litigious route is the least likely to obtain desired outcome and will be most time consuming and resource intensive. While the majority perceived direct negotiation to be the least time consuming and least expensive.
5. The study shows that the majority of respondents are keen to use mediation in the event of a conflict and are sufficiently interested to learn about mediation, even though mediation may not be the most resource efficient option to them.
6. The majority has no training in relation to management of conflict/dispute in the hospital setting (Question 23a) and are keen to learn more about it.

Based on the above observations, clinicians do see the advantages of mediation and that their desired outcome cannot be achieved by litigation if there is a material dispute with patients or their next-of-kin. Any further work to be done with clinicians may be in the direction of (1) increasing general understanding of mediation, (2) increasing awareness of avenues for mediation, and (3) become better aware of when to propose mediation. It is hoped that an increase in educational effort in these areas will improve the uptake of this dispute resolution tool. Nevertheless, the need for continued professional training in clinical care may reduce the priority in the learning of a non-medical related topic. The challenge therefore is how to build the mindset that learning to prevent or resolve disagreement is a key component of holistic patient care, as important as learning about the effects of the latest drug.

This study provided helpful insights into the knowledge, attitude and practice of mediation and dispute resolution in a public healthcare institution in Singapore. The knowledge base of the clinicians in this study about mediation was moderate and probably reflected the general lack of direct experience in the resolution of a dispute or training in mediation. This further corroborated with the general response that the uptake of mediation in the healthcare setting is currently poor in Singapore due to the lack of awareness and perceived lack of avenue among the surveyed clinicians. To help equip future generation of doctors with the necessary skills to navigate an increasingly litigious healthcare environment, medical students should be introduced to mediation and negotiation skills that have been tailored to the healthcare setting and introduced through existing modules relating to patient communication.

There are several limitations to this study. Firstly, this study was conducted in only one tertiary-care teaching hospital and the findings may not be easily generalisable to other clinical settings (e.g. primary-care). Secondly, the response rate from the clinicians is low, which may increase the risk of non-response bias. This may skew the overall responses towards respondents who already have an interest in mediation and have better knowledge about it. Consequently, the results obtained from this survey should be interpreted with care and should be seen as a 'best case' scenario. Nevertheless, the spread of the experience respondents is representative of the overall clinician population in our hospital and should provide a reasonably representative view of the clinicians in our institution.
