# Correction: A knowledge, attitude, and practice survey on mediation among clinicians in a tertiary-care hospital in Singapore

**DOI:** 10.1371/journal.pone.0205451

**Published:** 2018-10-04

**Authors:** Wenrong Lin, Zheng Jye Ling, Siqi Liu, Joel Tye Beng Lee, Marcus Lim, Aloysius Goh, Sean Lim, Peter George Manning, Mengling Feng, Tze Ping Loh

There are errors in the Funding and Competing Interests sections. The correct Funding statement is: Author Sean Lim currently receives salary from Peacemakers Consulting Services Pte Ltd, Singapore. The specific roles of these authors are articulated in the "author contributions" section. The funders of this study had no role in study design, data collection and analysis, decision to publish, or preparation of this manuscript. The correct Competing Interests statement is: Author Sean Lim is currently employed by Peacemakers Consulting Services Pte Ltd, Singapore. This does not alter our adherence to PLOS ONE policies on sharing data and materials. There are no patents, products in development or marketed products to declare.

There are errors in the author affiliations. The affiliations should appear as shown here:

Wenrong Lin^1^, Zheng Jye Ling^2^, Siqi Liu^3^, Joel Tye Beng Lee^4^, Marcus Lim^4^, Aloysius Goh^5^, Sean Lim^6^, Peter George Manning^7^, Mengling Feng^3^, Tze Ping Loh^7,8,9^

**1** Legal Office, National University Hospital, Singapore, **2** Academic Information Office, National University Hospital, Singapore, **3** Saw Swee Hock School of Public Health, National University of Singapore, Singapore **4** Singapore International Mediation Institute, Singapore, **5** Singapore International Mediation Centre, Singapore, **6** Peacemakers Consulting Services Pte Ltd, Singapore **7** Clinical Risk Management, National University Hospital, Singapore, **8** Department of Laboratory Medicine, National University Hospital, Singapore, **9** Biomedical Institute for Global Health Research and Technology, National University of Singapore, Singapore
